# Loss of partner and breast cancer prognosis — a population-based study, Denmark, 1994–2010

**DOI:** 10.1038/bjc.2012.96

**Published:** 2012-03-20

**Authors:** M H Olsen, P E Bidstrup, K Frederiksen, N H Rod, M Grønbæk, S O Dalton, C Johansen

**Affiliations:** 1 Survivorship, Danish Cancer Society Research Center, Strandboulevarden 49, 2100 Copenhagen, Denmark; 2 Department of Public Health, University of Copenhagen, Øster Farimagsgade 5, 1014 Copenhagen, Denmark; 3 National Institute of Public Health, University of Southern Denmark, Øster Farimagsgade 5A, 1353 Copenhagen, Denmark

**Keywords:** psychological stress, life change events, breast neoplasms, recurrence, mortality, prognosis

## Abstract

**Background:**

The extent to which experiencing a stressful life event influences breast cancer prognosis remains unknown, as the findings of the few previous epidemiological studies are inconsistent. This large population-based study examines the association between a common major life event, loss of a partner and breast cancer recurrence and all-cause mortality.

**Methods:**

*N*=21 213 women diagnosed with a first primary breast cancer 1994–2006, who had a cohabiting partner in the 4 years before their breast cancer diagnosis, were followed for death and recurrence in population-based registers and clinical databases. Information on education, disposable income, comorbidity and prognostic risk factors were included in Cox regression analyses.

**Results:**

Women who had lost a partner either before diagnosis or in subsequent years were not at significantly higher risk of recurrence or dying than women who had not lost a partner.

**Conclusion:**

Our results do not support the concern that experiencing a stressful life event, the loss of a partner, negatively affects prognosis of breast cancer.

A stressful life event may affect prognosis of breast cancer directly through stress-induced alterations of the immune and neuroendocrine system and indirectly through changes in health behaviour, such as physical activity, consumption of alcohol, compliance to therapy and coping with disease. In all, six previous studies have addressed the association between stressful life events and breast cancer prognosis: two of which found a significantly increased risk for recurrence (Ramirez *et al*, 1989; Palesh *et al*, 2007), two studies found no association (Hislop *et al*, 1987; Maunsell *et al*, 2001), whereas two studies found, contrary to what was expected, a significantly lower risk of recurrence (Barraclough *et al*, 1992; Graham *et al*, 2002). These inconsistent findings may be due to the use of different measures of exposure and methodological weaknesses including self-reported measure of exposure, small samples (*N*=94–665) and selection or recall bias (refer to web appendix: Supplementary Table A1). In this large population-based study, of more than 20 000 breast cancer cases, we use objective information from population-based registers and clinical databases to examine the association between a single life event stressor, loss of a partner and breast cancer prognosis. Loss of a partner is a common and also very stressful life event, implying considerable changes to everyday life (Holmes and Rahe, 1967).

## Materials and methods

### Linkage of registry data

Information on sex, date of birth, current and historical addresses, emigration, disappearance and death with date of these incidences were obtained from the Central Population Register in which all Danish residents since 1968 have been registered with a personal identification number allowing linkage of information between national registers (Pedersen *et al*, 2006).

### Breast cancer

We obtained information on date of breast cancer diagnosis (defined as date of primary surgery), date of recurrence (which is reported up to 10 years after diagnosis), tumour size (in mm), number of tumour-positive lymph nodes, malignancy grade, hormone receptor status and menopausal status, from Danish Breast Cancer Cooperative Group, which contain information on nearly 95% of all breast cancer cases in Denmark since 1977 (Møller *et al*, 2008).

### Other cancers

Information on first primary cancer, excluding nonmelanoma skin cancer, was obtained from the Danish Cancer Registry, which since 1943 have registered all cases of cancer (ICD-7) in Denmark (Storm *et al*, 1997).

### Stressful life event

A stressful life event was defined as the death of a cohabiting partner either in the 4 years before breast cancer diagnosis or in subsequent years. ‘Cohabitation’ was defined as two persons of the opposite sex over the age of 16, with a maximum age difference of 15 years, living at the same address with no other adults in the residence.

### Socioeconomic status and comorbidity

Information on educational level and disposable income (categorised in Table 1) was obtained 2 years before breast cancer diagnosis from the population-based Integrated Database for Labour Market Research in Statistics Denmark with data on sociodemographic factors in Denmark since 1980 (Thygesen, 1995).

Information on comorbidity categorised according to the Charlson comorbidity index (scores 0, 1 and ⩾2), excluding cancers (Charlson *et al*, 1987) was obtained from the Danish National Patient Register with information on all somatic diseases leading to hospitalisation since 1977, and from 1995 also information on all outpatient visits (Andersen *et al*, 1999).

### Analysed cohort

To attain accurate information from the included registers our base population is restricted to all 3.4 million Danish residents born between 1925 and 1973 who resided in Denmark 1994–2006 and entered the cohort at age 30 (for more details Dalton *et al*, 2008). From this population we identified 22 366 women diagnosed with breast cancer between 1 January 1994 and 31 December 2006 with no previous history of cancer (except nonmelanoma skin cancer), who resided in Denmark 2 years before diagnosis, and had a cohabiting partner up to 4 years before their breast cancer diagnosis. We excluded 1153 women with missing values on one or more covariates or who had resided in Denmark for less than 2 years. In all, 21 213 eligible women were followed for recurrence and death.

### Statistical analyses

We used Cox regression to assess hazard ratios (HRs) with 95% confidence intervals (CIs) for all-cause mortality and breast cancer recurrence, respectively, according to the vital status of the partner. For all-cause mortality follow-up time was counted from the date of diagnosis until death, emigration or 31 December 2010, whichever came first. For breast cancer recurrence follow-up time was counted from the date of diagnosis until death, emigration, 10 years of follow-up or 31 December 2006, whichever came first. The exposure, death of partner (after diagnosis), was included as a time-dependent variable, so that person–time before the death of the partner was counted as unexposed, whereas person–time after the date of death of the partner was counted as exposed. In all analyses, time since breast cancer diagnosis was used as the underlying time scale, and baseline hazards were allowed to vary across age at breast cancer diagnosis in 1-year intervals. The HRs were first adjusted for educational level and income, both considered as potential confounders. Subsequently, we adjusted for comorbidity, period of diagnosis, tumour size, number of positive lymph nodes, receptor status and malignancy grade (I–IV), as these factors are strongly associated with outcome, however, not obviously associated with the exposure. We estimated HRs in the intervals [0–1],]1–2],]3–4] years for exposure before diagnosis and for latencies of: [0–2],] 2–5],]5–17] years for exposure after diagnosis. We investigated whether a change in cohabitation status influenced the estimated association, by censoring at 1 January in the year in which the cohabitation status changed. Further, we estimated the association for women diagnosed with hormone-receptor-positive breast cancer and post-menopausal women only.

## Results

In the analysis of the association between loss of partner and all-cause mortality 172 773 person–years of follow-up were accrued, with a median follow-up of 7.7 years (ranging 0–17). During follow-up, 5660 women died, 762 lost their partner in the 4 years before their breast cancer diagnosis and 2259 lost their partner during follow-up. As expected, women who lost their partner were older and had a lower education and income compared with those who did not (Table 1). After adjustment for education and income as well as period of diagnosis, comorbidity and severity of breast cancer, women who had lost a partner were not at a significantly higher risk for recurrence or all-cause death from that of women who did not lose a partner, no matter if the event happened in the 4 years before diagnosis or in subsequent years (Table 2) or at different latencies (Table 3). We found only minor changes to the estimates when censoring at change in cohabitation status and when measuring the association only among women diagnosed with hormone-receptor-positive breast cancer or post-menopausal women (results not shown).

## Discussion

Our results do not support the concern that experiencing a major stressful life event, loss of a partner, negatively affects breast cancer recurrence or all-cause mortality, whether the event occurs in the 4 years before or 0–17 years after diagnosis. Of six previous studies four support this finding (Hislop *et al*, 1987; Barraclough *et al*, 1992; Maunsell *et al*, 2001; Graham *et al* 2002). Two previous studies found a higher risk of recurrence among women reporting stressful life events, however, both were of retrospective design and the observed estimate may reflect differential recall and reporting of events (Ramirez *et al*, 1989; Palesh *et al*, 2007).

We addressed several methodological limitations of the previous studies. First, we included more than 30 times as many cancer patients as the largest study published so far. Second, the use of national registers and databases to identify the study population ensures high representativeness and minimal risk for selection bias. Third, the exposure (death of partner) was measured independently of the participants, eliminating recall bias. Fourth, the register-based cohort design ensures temporality and minimises loss to follow-up and misclassification of outcomes. Finally, we estimated the effect of the death of partner before and after diagnosis on both recurrence and all-cause mortality.

Still, loss of partner is relatively rare among newly diagnosed breast cancer patients, and 73% of the cohort was alive and recurrence free at exit, resulting in small number of events in certain subgroups. The observed estimates of all-cause mortality may represent overestimates of breast cancer-specific mortality. We adjusted, however, the analyses for comorbidity and investigated also recurrence as outcome. Finally, an association may be present among women with poor coping resources or accumulated stressful life events. We were unable to take these into account.

The death of a partner is a common major life event, and our finding of no association with breast cancer recurrence or all-cause mortality may provide reassurance for women confronting breast cancer.

## Figures and Tables

**Table 1:** Descriptive characteristics at entry of 21 213 women with breast cancer diagnosed in 1994–2006, Denmark, by vital-status of the partner at exit

	**Partner's death before diagnosis (*n*=762), No. (%)**	**Partner's death after diagnosis (*n*=2259), No. (%)**	**Partner alive at exit (*n*=18 192), No. (%)**
*Sociodemographic characteristics*			
Age at time of diagnosis (years)			
30–39	2 (1)	25 (1)	1266 (7)
40–49	40 (5)	219 (10)	4640 (26)
50–59	154 (20)	670 (30)	6926 (38)
60–69	385 (51)	1061 (47)	4501 (25)
⩾70	181 (24)	284 (13)	859 (5)
Mean/median	64/65	60/61	54/54
			
Level of education^a^			
Basic or high school	440 (58)	1204 (53)	7127 (39)
Vocational education	227 (30)	703 (31)	6551 (36)
Higher education	95 (12)	352 (16)	4514 (25)
			
Disposable income^b^			
Lowest (1st quartile)	340 (45)	536 (24)	2489 (14)
Middle (2nd–3rd quartile)	308 (40)	1107 (49)	8468 (47)
Highest (4th quartile)	114 (15)	616 (27)	7235 (40)
			
*Medical characteristics*			
Tumour size (mm)			
0–10	98 (13)	415 (18)	2851 (16)
11–20	297 (39)	963 (43)	7352 (40)
21–50	311 (41)	780 (35)	6560 (36)
⩾51	22 (3)	53 (2)	737 (4)
Unknown	34 (4)	48 (2)	692 (4)
			
No. of positive lymph nodes			
0	393 (52)	1323 (59)	8979 (49)
1–3	204 (27)	644 (29)	5380 (30)
⩾4	140 (18)	274 (12)	3434 (19)
Unknown	25 (3)	18 (1)	399 (2)
			
Malignancy grade			
Grade I	186 (24)	660 (29)	4197 (23)
Grade II	261 (34)	738 (33)	6248 (34)
Grade III	130 (17)	321 (14)	3795 (21)
Grade IV	49 (6)	85 (4)	861 (5)
Unknown	136 (18)	455 (20)	3091 (17)
			
ER and PR Status^c^			
Negative	129 (17)	380 (17)	3882 (21)
Positive	588 (77)	1733 (77)	13 254 (73)
Unknown	45 (6)	146 (6)	1056 (6)
			
Period of diagnosis			
1994–1996	49 (6)	608 (27)	3100 (17)
1997–1999	134 (18)	557 (25)	3792 (21)
2000–2002	217 (28)	580 (26)	4527 (25)
2003–2006	362 (48)	514 (23)	6773 (37)
			
Charlson comorbidity index^d^			
0	603 (79)	1976 (87)	16 334 (90)
1	100 (13)	176 (8)	1201 (7)
⩾2	59 (8)	107 (5)	657 (4)

a Highest-education attained 2 years before diagnosis: basic school/high school (7–12 years of primary, secondary and grammar school); vocational training (10–12 years of education); higher education (⩾13 years of education).

b Disposable income extracted 2 years before diagnosis; income quartiles calculated according to the entire population in the investigated age group, after taxation and interest per person, adjusted for number of people in the household and deflated according to the 2000 value of the Danish crown, as: deflated household income/(no. of person in household 0.6).

c Negative indicates that individual was estrogen receptor (ER) and progesterone receptor (PR) negative. Positive indicates ER-positive or PR-positive.

d Accumulated value of all hospital contacts from 1978 to date of diagnosis; scores weighted by level of severity assigned to 19 conditions (excluding cancers) and grouped on the basis of the cumulated sum of scores of 0, 1 and ⩾2.

**Table 2:** Hazard ratios (HRs) for death and recurrence with 95% confidence intervals (CIs) according to partner's vital status among 21 213 women with breast cancer diagnosed in 1994–2006, Denmark

				**Crude** a		**Adjusted HR** b		**Adjusted HR** c	
	***N***	**Person years**	**Events**	**HR**	**(95% CI)**	**HR**	**(95% CI)**	**HR**	**(95% CI)**
*All-cause mortality*									
Partner alive at exit (ref.)	18 192	144 410	4977	1.00		1.00		1.00	
Exposed in the 4 years before diagnosis	762	5216	243	1.14	(1.00, 1.30)	1.07	(0.94, 1.22)	1.10	(0.95, 1.27)
Exposed 0–17 years after diagnosis	2259	23 147	440	1.12	(1.01, 1.25)	1.09	(0.98, 1.20)	1.09	(0.98, 1.22)
									
*Recurrence*									
Partner alive at exit (ref.)	19 312	86 453	2635	1.00		1.00		1.00	
Exposed in the 4 years before diagnosis	762	2839	59	0.83	(0.64, 1.08)	0.82	(0.63, 1.06)	0.82	(0.61, 1.09)
Exposed 0–17 years after diagnosis	1139	7581	85	0.98	(0.78, 1.22)	0.97	(0.78, 1.21)	0.93	(0.73, 1.18)

a Stratified by age at diagnosis in 1-year intervals.

b Adjusted for the highest-attained educational level, disposable income, and stratified by age at diagnosis in 1-year intervals (defined in Table 1).

c Adjusted for the highest-attained educational level, disposable income, period of diagnosis, comorbidity, tumour size, no. of tumour-positive lymph-nodes, hormone receptor status, malignancy grade and stratified by age at diagnosis in 1-year intervals (*N*=19 186) (defined in Table 1).

**Table 3:** Hazard ratios (HRs) for death and their 95% confidence intervals (CIs) according to partner's vital status and time between diagnosis, death and death of partner among 21 213 women with breast cancer diagnosed in 1994–2006, Denmark

				**Crude** a		**Adjusted HR** b		**Adjusted HR** c	
	**N**	**Person years**	**Events**	**HR** d	**(95% CI)**	**HR** d	**(95% CI)**	**HR** d	**(95% CI)**
*All-cause mortality*									
Exposure before diagnosis									
Time from bereavement to diagnosis (years)									
[0–1]	228	1610	76	1.17	(0.93, 1.48)	1.15	(0.91, 1.44)	1.04	(0.82, 1.34)
]1–2]	195	1391	65	1.14	(0.89, 1.46)	1.04	(0.81, 1.34)	1.05	(0.80, 1.38)
]2–4]	339	2215	102	1.11	(0.91, 1.36)	1.04	(0.85, 1.26)	1.19	(0.96, 1.47)
									
Exposure after diagnosis									
Time from bereavement to death (years)									
[0–1]	647	5768	173	1.20	(0.98, 1.48)	1.16	(0.95, 1.43)	1.11	(0.89, 1.39)
[1–5]	766	7237	156	1.11	(0.97, 1.28)	1.08	(0.94, 1.23)	1.09	(0.94, 1.26)
>5	846	10142	111	1.07	(0.88, 1.31)	1.04	(0.85, 1.27)	1.08	(0.87, 1.34)
									
*Recurrence*									
Exposure before diagnosis									
Time from bereavement to diagnosis (years)									
[0–1]	228	904	15	0.66	(0.40, 1.10)	0.66	(0.39, 1.09)	0.57	(0.32, 1.01)
[1–2]	195	800	14	0.71	(0.42, 1.20)	0.69	(0.41, 1.17)	0.74	(0.42, 1.32)
[2–4]	339	1135	30	1.05	(0.73, 1.51)	1.03	(0.72, 1.49)	1.11	(0.74, 1.65)
									
Exposure after diagnosis									
Time from bereavement to death (years)									
[0–1]	467	2439	48	0.90	(0.61, 1.34)	0.90	(0.60, 1.33)	0.73	(0.46, 1.17)
[1–5]	412	2837	28	1.01	(0.76, 1.34)	1.00	(0.75, 1.33)	1.01	(0.75, 1.36)
>5	260	2306	9	1.02	(0.52, 2.00)	1.02	(0.52, 2.00)	1.16	(0.57, 2.37)

a Stratified by age at diagnosis in 1-year intervals.

b Adjusted for the highest-attained educational level, disposable income and stratified by age at diagnosis in 1-year intervals.

c Adjusted for the highest-attained educational level, disposable income, period of diagnosis, comorbidity, tumour size, no. of tumour-positive lymph-nodes, hormone receptor status, malignancy grade and stratified by age at diagnosis in 1-year intervals (*N*=19 186) (defined in Table 1).

d The reference category (HR=1) are not experiencing the death of a cohabiting partner in the given year-interval.

## References

[bib1] Andersen TF, Madsen M, Joergensen J, Mellemkjoer L, Olsen JH (1999) The Danish National Hospital Register. A valuable source of data for modern health sciences. Dan Med Bull 46: 263–268 10421985

[bib2] Barraclough J, Pinder P, Cruddas M, Osmond C, Taylor I, Perry M (1992) Life events and breast cancer prognosis. BMJ 302: 1078–1081 10.1136/bmj.304.6834.1078 PMC1881923 1586819

[bib3] Charlson ME, Pompei P, Ales KL, MacKenzie CR (1987) A new method of classifying prognostic comorbiduty in longitudinal studies: development and validation. J Chron Dis 40: 373–383 3558716 10.1016/0021-9681(87)90171-8

[bib4] Dalton SO, Steding-Jesen M, Gislum M, Frederiksen K, Engholm G, Schüz J (2008) Social inequality and incidence of and survival from cancer in a population-based study in Denmark, 1994–2003: background, aims, material and methods. Eur J Cancer 44: 1938–1949 18684615 10.1016/j.ejca.2008.06.010

[bib5] Graham J, Ramirez A, Love S, Richards M, Burgess C (2002) Stressful life experiences and risk of recurrence of breast cancer: observational cohort study. BMJ 324: 1420–1423 12065263 10.1136/bmj.324.7351.1420 PMC115851

[bib6] Hislop TG, Waxler NE, Coldman AJ, Elwood JM, Kan L (1987) The prognostic significance of psychosocial factors in women with breast cancer. J Chronic Dis 40: 729–735 3597675 10.1016/0021-9681(87)90110-x

[bib7] Holmes TH, Rahe RH (1967) The social readjustment rating scale. J Psychosom Res 11: 213–218 6059863 10.1016/0022-3999(67)90010-4

[bib8] Maunsell E, Brisson J, Mondor M, Verreault R, Deschenes L (2001) Stressful life events and survival after breast cancer. Psychosom Med 63: 306–315 11292280 10.1097/00006842-200103000-00017

[bib9] Møller S, Jensen MB, Ejlertsen B, Bjerre KD, Larsen M, Hansen HB, Christiansen P, Mouridsen HT, Danish Breast Cancer Cooperative Group (2008) The clinical database and the treatment guidelines of the Danish Breast Cancer Cooperative Group (DBCG); its 30-years experience and future promise. Acta Oncol 47: 506–524 18465317 10.1080/02841860802059259

[bib10] Palesh O, Butler LD, Koopman C, Giese-Davis J, Carlson R, Spiegel D (2007) Stress history and breast cancer recurrence. J Psychosom Res 63: 233–239 17719359 10.1016/j.jpsychores.2007.05.012 PMC2094358

[bib11] Pedersen CB, Gøtzsche H, Møller JO, Mortensen PB (2006) The Danish Civil Registration System. A cohort of eight million persons. Dan Med Bul 53: 441–449 17150149

[bib12] Ramirez AJ, Craig TK, Watson JP, Fentiman IS, North WR, Rubens RD (1989) Stress and recurrence of breast cancer. BMJ 298: 291–293 2493899 10.1136/bmj.298.6669.291 PMC1835577

[bib13] Storm HH, Michelsen EV, Clemmensen IH, Pihl K (1997) The Danish Cancer Registry- history, content, quality and use. Dan Med Bull 44: 535–539 9408738

[bib14] Thygesen L (1995) The register-based system of demographic and social statistics in Denmark-an overview. Stat J UN Econ Commun Eur 12: 49–55 12320272

