# Supplementary material for: Loss of partner and breast cancer prognosis — a population-based study, Denmark, 1994–2010
Source: Br J Cancer. 2012 Mar 20;106(9):1560–3. doi: 10.1038/bjc.2012.96 (PMC3341857; doi:10.1038/bjc.2012.96)
Supplement: Supplementary Table [file bjc201296x1.doc]

| **Table A1** Characteristics of previous studies on the association between stressful life events and breast cancer prognosis. | | | | | | | | | | | | | |
| --- | --- | --- | --- | --- | --- | --- | --- | --- | --- | --- | --- | --- | --- |
| **Study**^1^ | | | **Design (follow-up)** | **Sample Size n** | **Exposure** | **Outcome** | | **Results** | | | **Possible Limitations** |  | |
| Hislop et al.,1987 | | | Cohort  (4 years) | 133 | ∑ Social Readjustment Rating Scale  (all life before diagnosis) | Recurrence and all-cause Mortality | | No association between ∑ of recent life change events and recurrence (p=0.48) or death (p=0.92) | | | Self-reported exposure, small sample size, risk of selection bias, no subjective impact of events, not cancer specific mortality |  | |
| Ramirez et al.,1989 | | Retrospective Case-control | | 100 | ∑ Bedford College life events and difficulties schedule  (all life before diagnosis) | | Recurrence | | Higher risk of recurrence among women reporting one or more stressful life event (RR=5.67; 95%CI 1.57;37.20) | Self-reported exposure, small sample size, risk of re-call and selection-bias, no subjective impact of events | | |  |
| Barraclough et al.,1992 | | Cohort (3.5 years) | | 204 | ∑ Bedford College life events and difficulties schedule  (the year before and 4,24,42 months after diagnosis) | | Recurrence | | Lower risk of recurrence among women reporting one or more severely stressful life events in the year before diagnosis (HR=0.43;95% CI 0.20;0.93), but  no association when exposed after diagnosis (HR= 0.88; 95% CI 0.48;1.64) | Self-reported exposure, small sample size, short follow-up, risk of selection bias, no subjective impact of events | | |  |
| Maunsell et al.,2001 | | Cohort  (5 years) | | 673 | ∑ Events / ∑events* perceived impact / ∑Social Readjustment Rating Scale  (5 years before diagnosis) | | Breast cancer specific and all-cause Mortality | | No association between the ∑ of stressful life events in the five years before diagnosis and survival (HR=0.99; 95%CI 0.70;1.38) / (HR=0.97; 95% CI 0.73-1.31) / (HR=1.04; 95%CI 0.78;1.31) | Self-reported exposure, small sample size, risk of selection bias | | |  |
| Graham et al.,2002 | | Cohort  (5 years) | | 202 | ∑ Bedford College life events and difficulties schedule  (In the year before / 5 years after diagnosis) | | Recurrence | | Lower risk of recurrence among women reporting one or more severely stressful life event in the 5 years after diagnosis (HR=1.01;95% CI 0.58;1.74) but no association in the 5 years before diagnosis (HR=0.52; 95% CI 0.29;0.95) | Self-reported exposure, small sample size, risk for selection bias, no subjective impact of events | | |  |
| Palesh et al. ,2007 | Retrospective Cohort | | | 94 | ∑ stressful or traumatic events  (All life before diagnosis) | | Recurrence | | Shorter disease-free interval among women reporting traumatic or stressful life events before diagnosis (p=0.02) | Self-reported exposure, small sample size, risk of re-call and selection-bias | | |  |
| Olsen et al., 2011 | Cohort  (up to 12 years) | | | 21 213 | Death of cohabiting partner (In the 4 years before or up to 17 years after diagnosis) | | Recurrence and all-cause mortality | | No association between the death of a cohabiting partner and recurrence or all-cause mortality | Only single event, no subjective impact of events, not cancer specific mortality | | |  |

^1^ Studies were selected if analysing stressful life events as exposure and recurrence or survival as outcome. Studies were identified through searching the Medline database using the terms: (("Stress, Psychological" OR “life events” OR “Life Change Events”) AND (Survival OR Relapse OR Recurrence) AND (("Breast Neoplasms") OR ("breast Cancer") OR ((Breast OR mamma) AND (cancer OR Neoplasms))).

∑: the summation of events or item-scale
